# Supplementary material for: Fetal and postnatal metal dysregulation in autism
Source: Nat Commun. 2017 Jun 1;8:15493. doi: 10.1038/ncomms15493 (PMC5461492; doi:10.1038/ncomms15493)
Supplement: Supplementary Information — Supplementary Figures and Supplementary Methods. [file ncomms15493-s1.pdf]

Supplementary Methods: Detailed Statistical Methods

A variation of distributed lag models (DLMs) was used to detect critical windows of development using the data generated by tooth matrix biomarkers. The general strategy for DLMs yields an estimate of the effect of exposure incurred at a specific time window while adjusting for exposures at other times, under the assumption that the effect of exposure varies smoothly over time. Following methods developed by Coull and colleagues (Harvard University), we interchange the role of the outcome and exposure, in a reverse DLM, and use a functional spline model with time-varying coefficients to evaluate the association between a univariate continuous outcome Y_i (e.g., severity measured by ADOS-2) and a single exposure measured repeatedly, to accommodate the varying time points evaluated per tooth:

$$X_i(t) = \beta_0(t) + \beta_1(t)Y_i + \gamma(t)z_i + u_i + \varepsilon_{it} \qquad (1)$$

where $X_i(t)$ are the adjusted log-transformed metal concentrations over time, with covariates defined in z_i. The time varying coefficients are the intercept, $\beta_0(t)$, and estimate of the time varying correlation between X and Y, $\beta_1(t)$, with both Y and X centered and scaled. For example, when X and Y are standardized to have mean 0 and standard deviation of 1, the estimates for $\beta_1(t)$ are time-varying correlations between X and Y (see Supplementary Figure 1A where negative correlation is estimated from 10 weeks before birth and positive associations are estimated starting from 10 weeks postnatal). The random effect term, *u*, permits the assumption of a compound symmetry correlation pattern for intra-subject observations. A similar analysis strategy can be used with case-control data. When Y is a binary variable, $\beta_1(t)$ is the time-varying difference in standardized concentrations of X. That is, if there is evidence that the concentrations are higher among the cases at given time points compared to control, then an adverse association is claimed at those time points; if there is evidence that the concentrations are lower among the cases, then a deficit association is claimed.

Confidence Intervals:

In the reverse DLM, the 'sample size' includes the total number of measurements on each tooth for all subjects where it is reasonable to assume observations across subjects are independent and those within subjects are correlated. 95% confidence intervals at each time point are adjusted for the variability in the predicted model (Supplementary Figure 1B, here unadjusted for the random effect) and due to the intra-subject correlations (wider band in Supplementary Figure 1C in light blue to which our DLM spline is centered). However, these are piecewise 95% confidence intervals at specific time points and not a simultaneous 95% confidence band. To adjust for multiple testing, we overlay Holm-Bonferroni-adjusted 95% confidence intervals for $\beta_1(t)$ at regular intervals (Supplementary Figure 1D). To illustrate, consider a test of significance at every 5th week between -20 and 30 weeks around birth, for a total of 11 tests. A test statistic for the significance of $\beta_1(t)$ is constructed at each time point and the absolute values of the resulting test statistics are ranked from smallest to largest: i.e., $r_{[1]}$, $r_{[2]}$, …, $r_{[M]}$. A $100(1-\alpha/M)\%$ confidence interval is constructed for the time point with the largest test statistic $r_{[M]}$; a $100(1-\alpha/(M-1))\%$ confidence interval is constructed for the second largest test statistic $r_{[M-1]}$; …; and the time point with the smallest test statistic $r_{[1]}$ has a $100(1-\alpha)\%$ confidence interval. Comparisons are made in order from largest to smallest test statistics to accommodate a family-wise 95% confidence interval for the set of comparisons. Pre- and post-natal concentrations at time points where 0 is not included in the corresponding confidence interval are claimed to be associated with the response variable in later life.

The extension to the current analysis and the standard approach described above is that our data are based on twin pairs. Thus, $X_i(t)$ in our analyses is the difference in the log concentrations within the ith twin pair where, for the discordant pairs, the concentration from the healthy twin is subtracted from the ASD twin. So X values greater than 0 represent time points where the case has higher concentrations than the control. The twins are randomly ordered in the healthy concordant pairs for the subtraction.

We are interested in determining critical windows when the discordant pairs are different from differences in healthy or concordant twin pairs. The Y variable in our model for equation (1) is an indicator of whether the twin

pair is discordant or not. Specifically, the $\beta_1(t)$ in model (1) is the smoothed mean differences in concentrations from discordant pairs minus from concordant pairs (non-ASD control twins), i.e., $(x_{Case} - x_{Control}) - (x_{twin1} - x_{twin2})$. Thus, for example, when concordant pairs are similar and discordant cases have higher concentrations than the paired controls the beta coefficient is positive.

All models were adjusted for covariates including sex, zygosity, gestational age, the average birth weight of the twin pairs, and the standard deviation of the birth weight in the twin pairs.

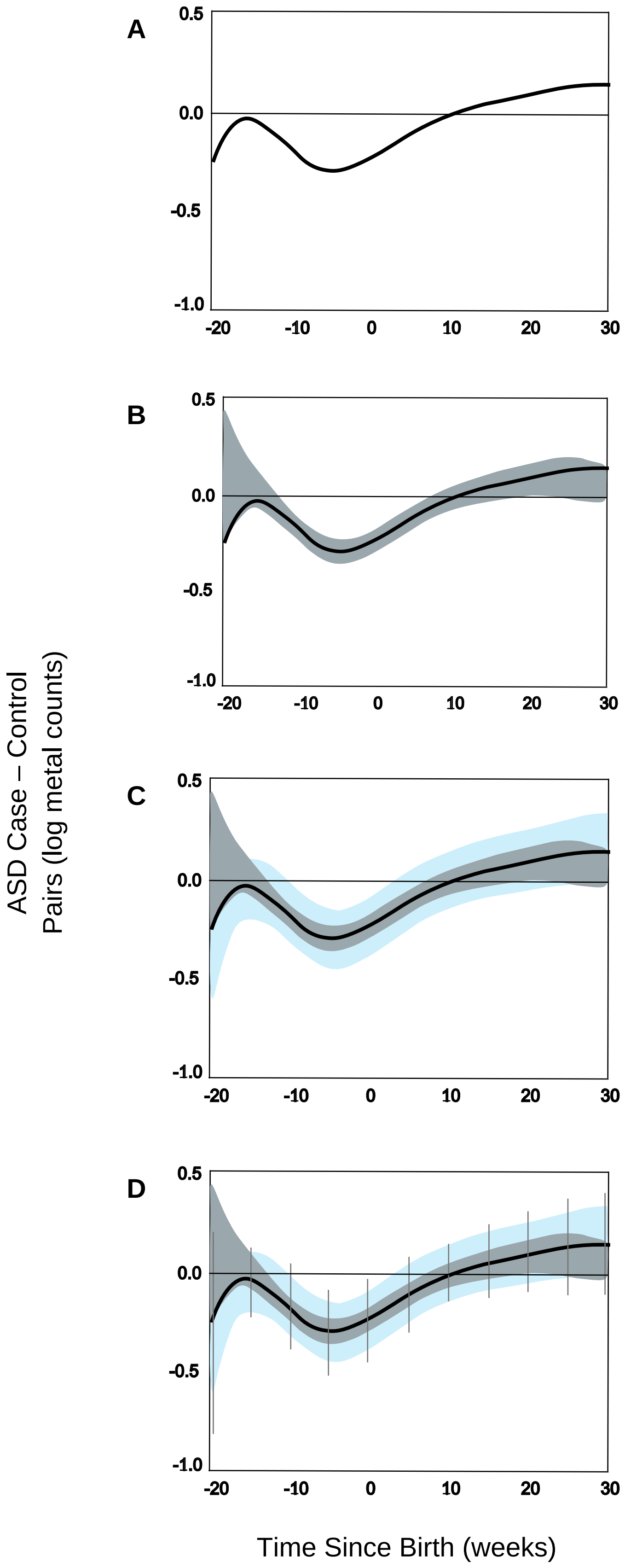

Supplementary Figure 1. Key steps in the construction of the distributed lag models (DLMs) used in this study.

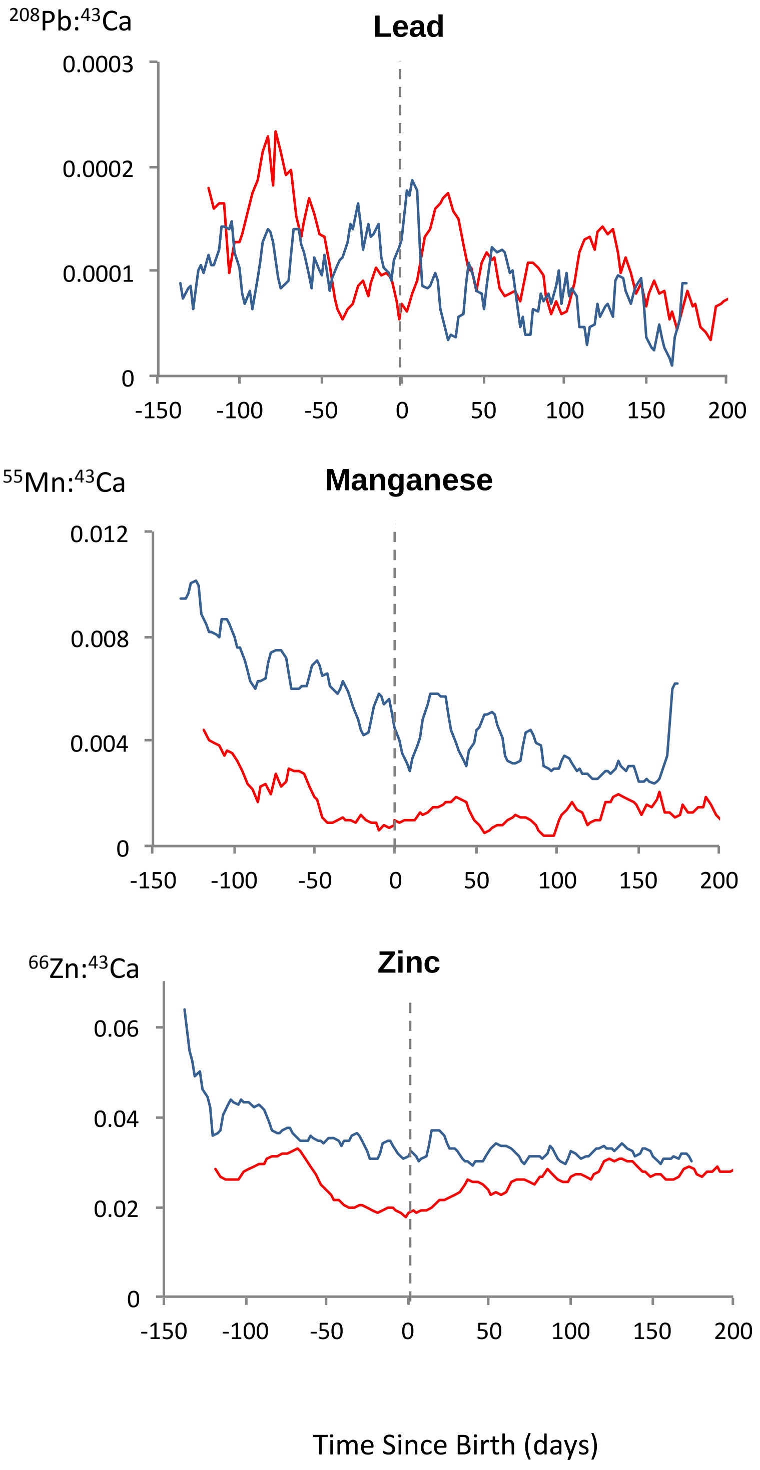

Supplementary Figure 2. Pre- and postnatal differences in metal uptake in an ASD discordant twin pair from a clinic in the United States. Consistent with the results from the RATSS study in Sweden (see Figures 3 – 5), zinc levels were lower in the case prior to birth and manganese levels were lower throughout the study period. Lead levels were not appreciably higher in the case, which is in contrast to the trends observed in the Swedish participants.

Pb

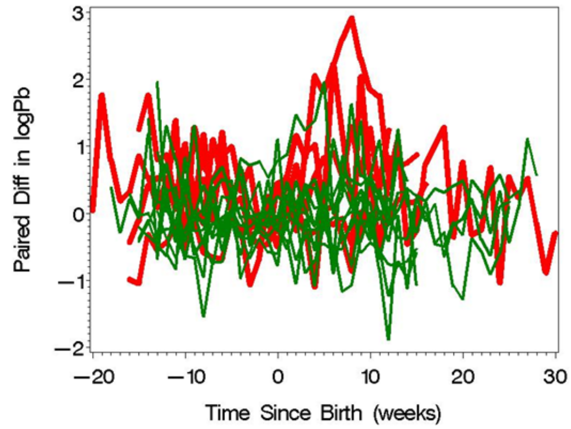

Zn

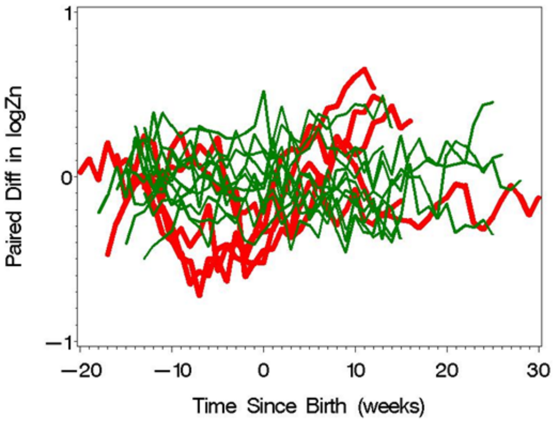

Ba

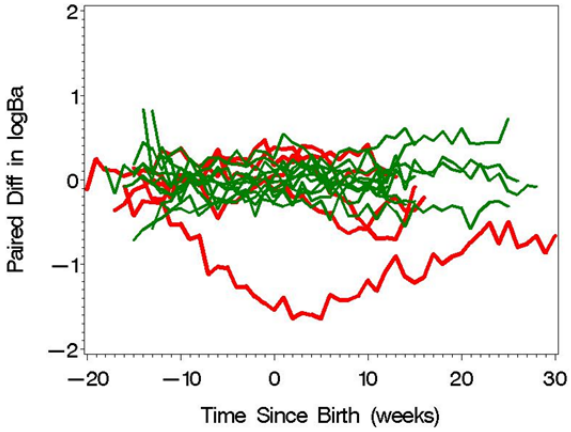

Cr

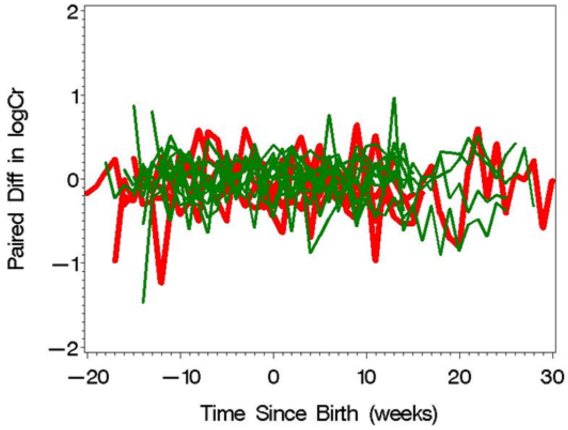

Cu

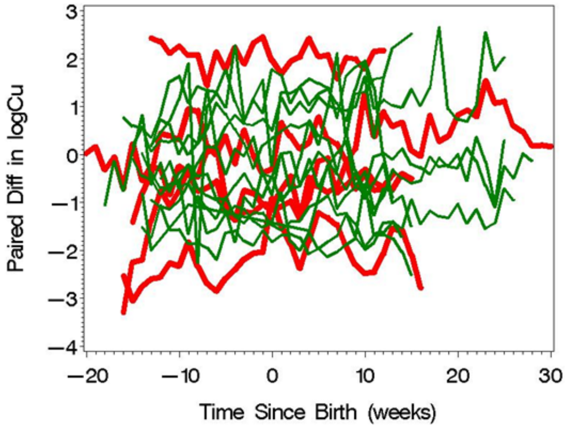

Li

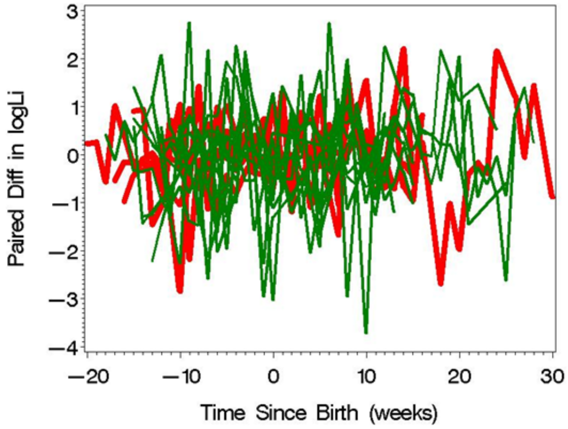

Mg

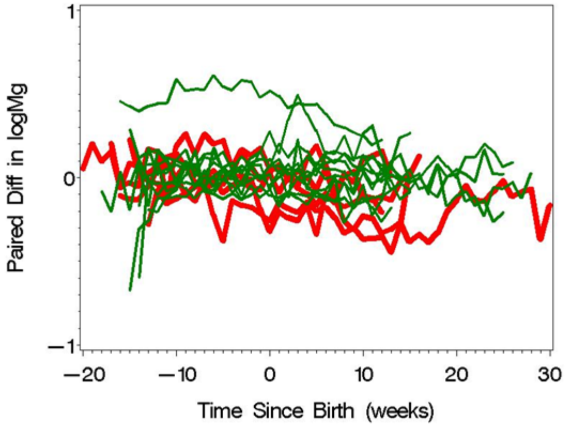

Mn

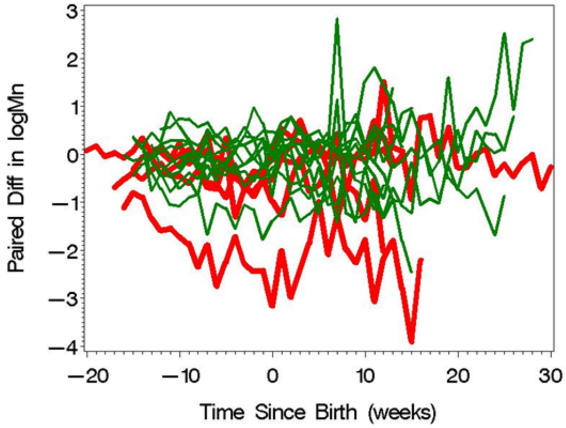

Sr

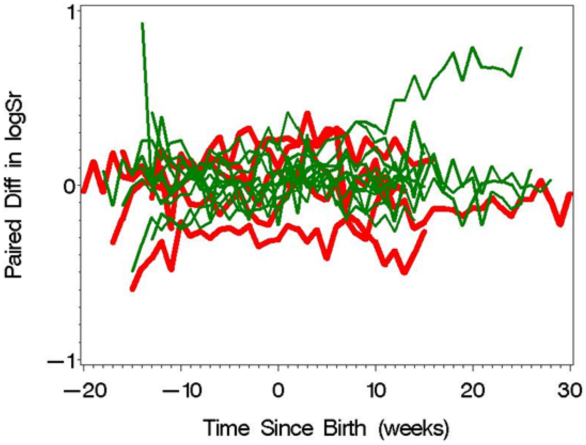

Sn

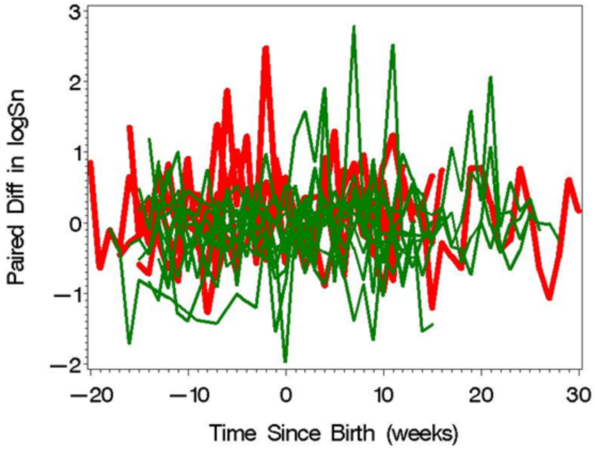

Supplementary Figure 3. Paired differences in log concentrations of 10 metals (Ba, Cr, Cu, Li, Mg, Mn, Pb, Sn, Sr, and Zn) between twins who are discordant for ASD (red; 3MZ; 4DZ) or both are controls (green; 9MZ; 10DZ).

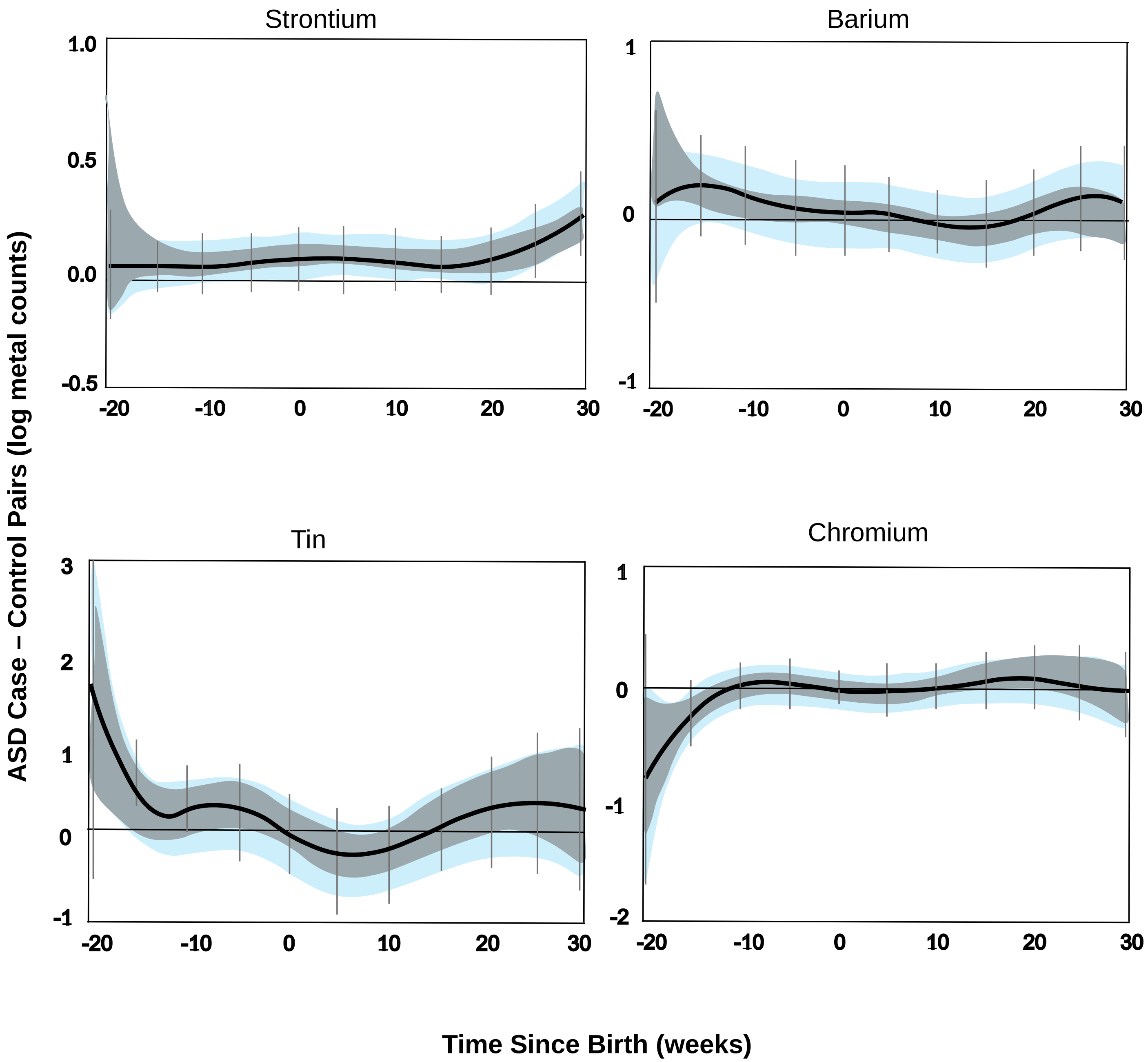
Strontium
Barium
Tin
Chromium
ASD Case – Control Pairs (log metal counts)
Time Since Birth (weeks)
1.0
0.5
0.0
-0.5
1
0
-1
3
2
-2
-20
-10
10
20
30

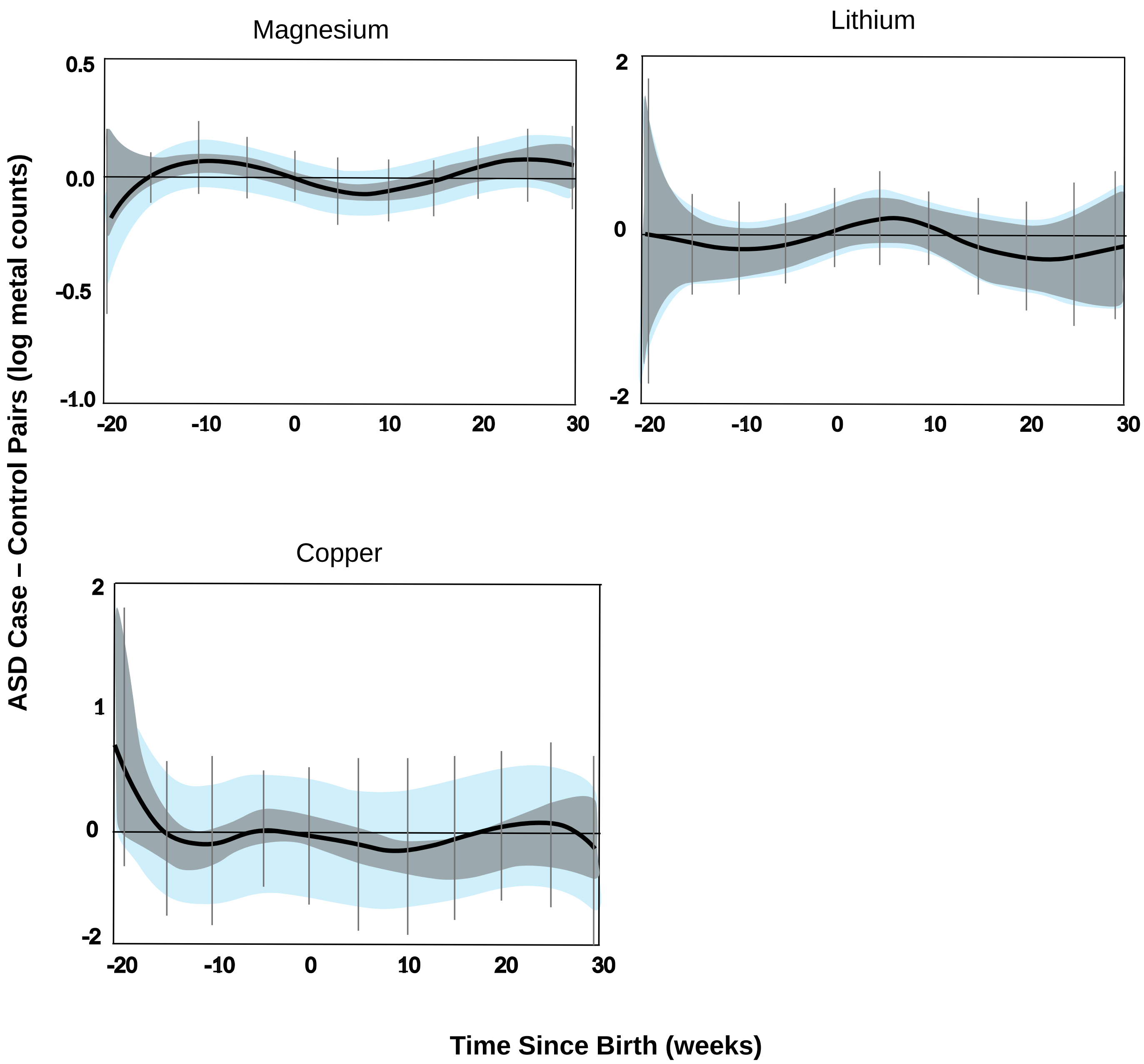

Supplementary Figure 4. Paired differences of barium (Ba); chromium (Cr); copper (Cu); lithium (Li); magnesium (Mg); strontium (Sr); and tin (Sn) between twin pairs discordant for ASD (3MZ; 4DZ) and healthy twin pairs (9MZ; 10DZ), adjusted for sex, zygosity, gestational age, birth weight (average and standard deviation in the pair). The grey bands are unadjusted for twin-pairs; the wider blue bands are adjusted for intra-pair correlations. Vertical grey lines are Holm-Bonferroni-corrected family-wise 95% confidence intervals for comparisons at every 5th week (i.e., 11 comparisons).
